# Immune protection of chickens conferred by a vaccine consisting of attenuated strains of *Salmonella* Enteritidis, Typhimurium and Infantis

**DOI:** 10.1186/s13567-016-0371-8

**Published:** 2016-10-15

**Authors:** Karolina Varmuzova, Marcela Faldynova, Marta Elsheimer-Matulova, Alena Sebkova, Ondrej Polansky, Hana Havlickova, Frantisek Sisak, Ivan Rychlik

**Affiliations:** Veterinary Research Institute, Hudcova 70, 621 00 Brno, Czech Republic

## Abstract

The colonization of poultry with different *Salmonella enterica* serovars poses an issue throughout the world. In this study we therefore tested the efficacy of a vaccine consisting of attenuated strains of *Salmonella enterica* serovars Enteritidis, Typhimurium and Infantis against challenge with the same serovars and with *S*. Agona, Dublin and Hadar. We tested oral and aerosol administration of the vaccine, with or without co-administration of cecal microbiota from adult hens. The protective effect was determined by bacterial counts of the challenge strains up to week 18 of life and by characterizing the immune response using real-time PCR specific for 16 different genes. We have shown that a vaccine consisting of attenuated *S*. Enteritidis, *S*. Typhimurium and *S*. Infantis protected chickens against challenge with the wild type strains of the same serovars and partially protected chickens also against challenge with isolates belonging to serovars Dublin or Hadar. Aerosol vaccination was more effective at inducing systemic immunity whilst oral vaccination stimulated a local immune response in the gut. Co-administration of cecal microbiota increased the protectiveness in the intestinal tract but slightly decreased the systemic immune response. Adjusting the vaccine composition and changing the administration route therefore affects vaccine efficacy.

**Electronic supplementary material:**

The online version of this article (doi:10.1186/s13567-016-0371-8) contains supplementary material, which is available to authorized users.

## Introduction

Although the incidence of human salmonellosis is gradually decreasing in the EU, *Salmonella enterica* is still one of the most frequent causative agents of gastroenteritis in humans worldwide [1]. Since the major reservoirs of *Salmonella enterica* for human populations are found in poultry flocks [2], it is expected that a decrease in *Salmonella* prevalence in poultry will also result in a decrease in the incidence of human salmonellosis.

One of the most feasible ways of reducing *Salmonella* prevalence in poultry flocks is vaccination. However, current live attenuated vaccines and vaccination schemes, though effective, exhibit several limitations. Live commercial vaccines for poultry are available for *S*. *enterica* serovars Enteritidis, Typhimurium and Gallinarum only, whilst there are over 2600 serovars of *Salmonella enterica* able to cause disease [3] and contradicting data have been reported on the protection of chickens vaccinated and challenged with different serovars [4–7]. Another issue in poultry production is that live *Salmonella* vaccines are usually administered via drinking water and due to the logistics in commercial poultry production, chickens are vaccinated after being transported from hatcheries to farms, i.e. at the age of 2 or 3 days. There is therefore no protection during the first 48–72 h of life despite the fact that chickens are the most sensitive to *Salmonella* infection immediately after hatching [8], and an immediate vaccination after hatching could partially protect chickens by growth inhibition of closely related *Salmonella* strains [9].

Rapid vaccination soon after hatching can be achieved by spraying. Although there are several reports on aerosol administration of live *Salmonella* vaccines to chickens [10–12], there is no data on the long term protection following this mode of vaccine administration. Moreover, chickens vaccinated by aerosol in these studies were challenged orally and although oral challenge test the induction of gut immunity, it may not be sufficient for the characterization of systemic immunity. Finally, since multiple tissues are stimulated following aerosol vaccination (i.e. respiratory tract and conjunctiva), a less localized and more systemic immune response can be expected.

Specific immunity requires approx. 2 weeks to develop. Although a certain degree of protection can be achieved by mere *Salmonella*–*Salmonella* competition, such protective potential is usually decreased in attenuated vaccine strains [13]. Independent experiments showed that chickens can be protected against *Salmonella* infection by providing them with microbiota from adult hens [14, 15], i.e. before the onset of specific immunity due to the vaccination. A combination of both approaches would result in immediate protection by microbiota followed by a specific immune response due to the vaccination. This has been tested successfully, although wild type *S*. Typhimurium, i.e. not an attenuated strain, was used for the immunization [16]. Moreover, with increasing knowledge of chicken microbiota composition and function [17, 18], the combination of vaccination and microbiota administration may represent a field worth pursuing.

In this study we therefore constructed a vaccine consisting of attenuated strains of three different serovars including *S*. Enteritidis, *S*. Typhimurium and *S*. Infantis. The vaccine was provided to chickens orally and by aerosol, with or without supplementation with gut microbiota and subsequently we tested and compared its protective capacity against challenge with homologous and heterologous *Salmonella* serovars.

## Materials and methods

### Bacterial strains

*S*. Enteritidis 147, *S*. Typhimurium 16E5 and *S*. Infantis 18G6 were used in this study. *S*. Enteritidis is a poultry isolate of phage type 4 with proven virulence for chickens and mice [19, 20]. *S*. Typhimurium is an isolate of phage type DT104 but without the multidrug resistance genomic island SGI1 [21]. *S*. Infantis has been selected as representing the most frequent *S*. Infantis clone detected in our previous study [22]. Clones sensitive to all commonly used antibiotics were used for the generation of attenuated mutants though for the challenge we used the same strains selected as spontaneously resistant to nalidixic acid. *S*. Derby, *S*. Hadar and *S*. Agona originated from laboratory collection of different *Salmonella* isolates resistant to antibiotics including nalidixic acid. All the strains were grown statically in LB broth or LB agar plates for 18 h at 37 °C.

The deletions in *S*. Enteritidis, Typhimurium and Infantis were produced as follows. First, the whole *Salmonella* pathogenicity island 1 (SPI1) was replaced with a kanamycin resistance gene cassette by λ red recombination, as described previously [23]. In the case of *S*. Enteritidis and *S*. Typhimurium, SPI1::Kan mutation was transduced with P22 phage into a fresh wild type strain. Since P22 does not propagate in *S*. Infantis, in this serovar we proceeded directly with the SPI1::Kan mutant generated by λ red recombination. This first step resulted in deletion of SPI1 but also served as a transient introduction of kanamycin resistance which was used as the selection marker during further manipulations. The remaining deletions were generated using overlap PCR to construct sequences with deletions [24]. The PCR products were cloned into pDM4 plasmid [25] and the plasmid was transferred by conjugation into the appropriate *Salmonella* strain, selecting for kanamycin (selection marker of SPI1::Kan mutants) and chloramphenicol (selection marker of pDM4) resistant clones, and selecting for chloramphenicol sensitive clones following growth of the recombinants in the presence of 5% sucrose [25]. In this way, *lon*, *fliC* and *fljB* (in the case of *S*. Typhimurium and *S*. Infantis) genes from start codon to stop codon were removed. Finally a “deleted” PCR product spanning SPI1::Kan sequence was generated, cloned into pDM4 and conjugated into *Salmonella* strains. Final recombinants were then selected as sensitive both to chloramphenicol and kanamycin. All the deletions were verified by PCR and finally, genomic sequences of *S*. Enteritidis, *S*. Typhimurium, *S*. Infantis wild type strains and their deletion mutants (without gap closures) were determined using NextSeq sequencing to confirm the deletions and exclude any unexpected genomic rearrangements.

### Experimental animals

Male, newly-hatched ISA Brown chickens (Hendrix Genetics, the Netherlands) were used in this study. The chickens were reared in perforated plastic boxes with free access to water and feed. Each of the experimental or control groups was kept in a separate room. All the experiments were approved by the Committee for Animal Welfare of the Ministry of Agriculture of the Czech Republic under permit number MZe1480.

### Experimental design

In the first experiment we tested the protective effect against challenge with homologous serovars. Forty-two chickens were orally administered a mixture containing 1 × 10^6^ CFU of attenuated *S*. Enteritidis, *S*. Typhimurium and *S*. Infantis strains in 0.1 mL of inoculum on day 1 of life and an additional 36 chickens served as non-vaccinated controls. On day 21, six vaccinated chickens were sacrificed to check for residual colonization of the vaccine strains and the remaining chickens were divided into 6 groups of 12 birds each. The vaccinated chickens, as well as the non-vaccinated chickens, were challenged with 3 × 10^7^ CFU of wild type *S*. Enteritidis, *S*. Typhimurium or *S*. Infantis in 0.1 mL of inoculum, respectively. Six chickens from each group were humanely euthanized 4 and 14 days post infection (dpi) and *Salmonella* counts in the cecum and liver were determined.

In the second experiment we tested protection against challenge with heterologous serovars *S*. Hadar, *S*. Agona and *S*. Dublin. The experiment was performed exactly as described above except that we included one more group which was challenged with *S*. Enteritidis to allow for a control with results from the previous “homologous challenge” experiment and on day 21, 12 vaccinated chickens were sacrificed to check for residual colonization of the vaccine strains.

Since in the initial vaccination experiments we observed that vaccine strains were present in the chickens on day 21 prior to the challenge, in the next experiment we tested shedding of the vaccine strains. Six chickens were orally vaccinated with 1 × 10^6^ CFU of the mixture of attenuated *S*. Enteritidis, *S*. Typhimurium and *S*. Infantis strains in 0.1 mL of inoculum on day 1 of life and revaccinated on day 21. Cloacal swabs were taken from all these chickens in weekly intervals from week 6 to week 14 of life.

In the last experiment we addressed to what extent the mode of vaccine administration and formulation would affect long-term protection. The first group of chickens served as a non-vaccinated control. Chickens in the remaining three groups were vaccinated on the day 1 of life. Chickens in group 2 were orally vaccinated with a mixture of attenuated *S*. Enteritidis, *S*. Typhimurium and *S*. Infantis. Group 3 was orally vaccinated with the vaccine strains mixed with an equal volume of cecal extract (referred to as cecal microbiota) from 34-week-old healthy hens. The cecal extract was prepared by pooling an equal amount of cecal contents of 3 donor hens and resuspension of 0.5 g of the cecal content in 5 mL of PBS with 1 mM cysteine. Following centrifugation at 500*g* for 1 min, the supernatant was collected and mixed with an equal volume of vaccine strains. In the last group of chickens, vaccine strains were administered by coarse aerosol application. The chickens were revaccinated orally on week 3 and 12 of life with the mixture of three vaccine strains and the revaccinations were performed in all vaccinated groups in the same way, i.e. irrespective of the first way of vaccine administration. When the chickens were 6 and 18 weeks old, six chickens from each group were challenged with *S*. Enteritidis, three of them orally and the other three intravenously. All the chickens were sacrificed 4 days later and *S*. Enteritidis counts were determined in the cecum and liver. In addition, three non-vaccinated and non-infected chickens were sacrificed.

### Bacteriology

After necropsy, approximately 0.5 g of liver tissue and cecal contents were homogenized in peptone water and tenfold serial dilutions were plated on XLD agar plates (HiMedia) supplemented with 20 µg/mL nalidixic acid for *Salmonella* enumeration. Samples negative for *Salmonella* after direct plating were subjected to enrichment in modified semi-solid Rappaport-Vassiliadis medium (Oxoid) for qualitative *Salmonella* determination. Counts positive for *Salmonella* after direct plating were logarithmically transformed. Samples positive only after enrichment were assigned a value of one and negative samples were assigned a value of zero.

### RNA purification, reverse transcription and real-time PCR

Liver and cecal tissue was collected into RNALater in the experiment with aerosol vaccination and stored at −80 °C. Total RNA was isolated with RNeasy Mini Kit (Qiagen), the concentration and purity of RNA was determined spectrophotometrically (Nanodrop, Thermo Scientific) and 1 µg of RNA was immediately reverse transcribed into cDNA using M-MLV reverse transcriptase (Invitrogen) and oligo dT primers. After reverse transcription, the cDNA was diluted 10 times with sterile water and kept at −20 °C prior to real-time PCR.

Quantitative real-time RT-PCR (qRT-PCR) was used to determine gene expression. Gene expression in the cecum was determined for AH221, AVD, CSF3, ES1, EX-FABP, IL-22, IL4I1, IL8, INFγ, iNOS, IRG1, LYG2, MMP7, MRP126, SAA and TRAP6 and gene expression in the liver was characterized by the expression of a slightly different subset comprising AVD, CSF3, EX-FABP, IgG, IL4I1, IRG1, MRP126, PTGDS, SAA, SCYA4, serpinB10, TGM4 and TRAP6. A list of all the primers together with a brief description of each gene function can be found in the Table 1 or recent review [26]. qRT-PCR was performed in 3 μL volumes in 384-well microplates using QuantiTect SYBR Green PCR Master Mix (Qiagen) and a Nanodrop II Stage pipetting station (Innovadyne, Farnborough, UK) for PCR mix dispensing. The amplification of PCR products and signal detection were performed using a LightCycler II (Roche, Basel, Switzerland) with an initial denaturation at 95 °C for 15 min followed by 40 cycles of 95 °C for 20 s, 60 °C for 30 s and 72 °C for 30 s. Each sample was subjected to qRT-PCR in duplicate and the mean Ct value of duplicates was used for subsequent calculations. The Ct values of the genes of interest were normalized (ΔCt) to an average Ct value of three house-keeping genes (GAPDH, TBP and UB) and the relative expression of each gene of interest was calculated as 2^−ΔCt^.

**Table 1:** **List of primers used in this study**

Gene	Gene function	Orient	Primer 5′–3′
AH221	CC type chemokine	For	CTCTGCTCCTCGGCTGTG
		Rev	TCCTTCCCTTTCTTGGTCAC
AVD	Avidin	For	CCTTTGGCTTCACTGTCAAT
		Rev	GCGAGTGAAGATGTTGATGC
CSF3	Colony stimulating factor 3	For	AACCTCTCCTCCAACATCCAG
		Rev	GTACGCCGTCTCCAGGAAG
ES1	ES1 protein homolog, mitochondrial-like	For	GGTGTACGATGGCAGTGAGAT
		Rev	CTCTGGCTATTCTGGCACTTTC
ExFABP	Extracellular fatty acid binding protein	For	GGAACTACACGGATGAGATGGT
		Rev	TGGCACATTAGTCTTGCTTTGT
IgG	IgG (IgY) immunoglobulin	For	GGGAGAAGAGTGGGAACCTC
		Rev	ATAACCAATCCTGGGTGCTG
IL22	Interleukin IL-22	For	CAGGAATCGCACCTACACCT
		Rev	TCATGTAGCAGCGGTTGTTC
IL4I	IL4-inducible gene	For	GGAGAAGGACTGGTATGTGGAG
		Rev	GCTTCAGGTCAAACTGCCTTAT
IL8	Interleukin IL-8	For	CAAGCCAAACACTCCTAACCAT
		Rev	AGCTCATTCCCCATCTTTACC
INFγ	Interferon γ	For	GCC GCA CATCAAACACATATCT
		Rev	TGAGACTGGCTCCTTTTCCTT
iNOS	Inducible NO synthase	For	GAACAGCCAGCTCATCCGATA
		Rev	CCCAAGCTCAATGCACAACTT
IRG1	Immune responsive gene 1	For	TCGTCGAAATCCATTGAGTG
		Rev	ACCGAGGTCTGCCAGAAAGT
LYG2	Lysozyme G2	For	GGGCACGAGAATACTTATTGACA
		Rev	TCATTGCTGTAGTCATCATGGAG
MMP7	Matrix metalloproteinase 7	For	GATGATGCAATTAGAAGGGCTTT
		Rev	CCACCTCTTCCATCAAAAGGATA
MRP126	Protein MRP-126	For	TGAAGCTCTTGATTGAGAAGCA
		Rev	CGAGATCCTTGAAGATTTGGTC
PTGDS	Prostaglandin D2 synthase	For	CATTCCTGTGCAAGCTGACTT
		Rev	CTGTTCCTCTTCTCGCACTGTT
SAA	Serum amyloid A	For	GCTTCGTGTTGCTCTCCATT
		Rev	TAGTTTGCCTCACGCATGTC
SCYA4	Small inducible cytokine A4, MIP-1β	For	TCATGCTGGTGTTGTGTTCA
		Rev	GGTGCATCAGTTCAGTTCCA
SERPINB10	Serine protease inhibitor	For	AGACTCAGGTCTTCTCTCTCACG
		Rev	TGTTGGTCTCATTCAGCTTGTT
TGM4	Transglutaminase 4	For	GCCTTCAACATACACAGCAAAC
		Rev	CAGACATGGCTCTGGATACAAC
TRAP6	Trappin 6	For	CACGGGGACACAGGCACCCTT
		Rev	CCACCCACCATCCCCTTGTCC

### Statistical analysis

A *t* test was used for the comparison of *Salmonella* counts in the liver comparing the counts in the vaccinated and non-vaccinated chickens after the challenge with the same serovar. In the experiment with oral and aerosol vaccination, ANOVA followed by Tukey’s test was used. χ^2^ test was used for the comparison of *Salmonella* presence or absence in the ceca of vaccinated and non-vaccinated chickens. All calculations were performed in Statistica v.9.1 software (StatSoft Inc., Tulsa, USA), except for PCA analysis which was performed in R programming language. Differences with *P* < 0.05 were considered as significant.

## Results

### Vaccination strains

The SPI1-*lon*-*fliC* mutant of *S*. Enteritidis and SPI1-*lon*-*fliC*-*fljB* mutant of *S*. Infantis grew in the form of mucoid colonies. This mucoid phenotype was not observed in the SPI1-*lon*-*fliC*-*fljB* mutant of *S*. Typhimurium. Full genome sequencing confirmed the presence of all mutations as designed and the absence of any additional difference between the wild type strain and appropriate mutant. Full genome sequencing also showed that both the wild type and mutant *S*. Typhimurium contained a 3281 bp deletion flanked by 10 bp AATGCGCTGG direct repeat. This deletion comprised a 3′ end of the *yojN* gene, whole *rcsB* and a 5′ end of the *rcsC* gene explaining the absence of the mucoid phenotype in *S*. Typhimurium despite the *lon* gene deletion [27].

### Protection against homologous challenge

When 6 chickens were examined for the presence of the vaccine strains on day 21, i.e. just before challenge, all 6 chickens were positive for *Salmonella* in the cecum and 4 out of 6 chickens were positive also in the liver. Out of 56 randomly picked colonies, 2 belonged to serovar Enteritidis, 11 to Typhimurium and 43 to serovar Infantis.

**Figure 1:**
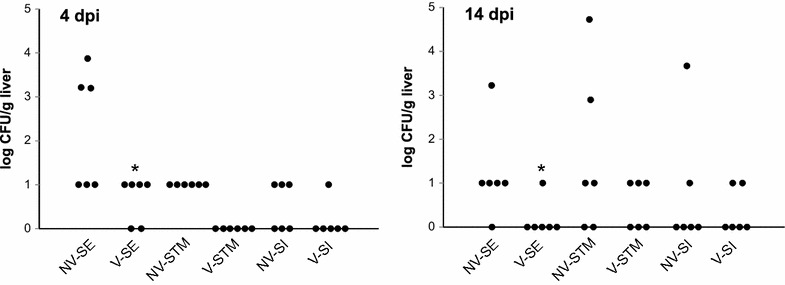
**Protective effect of** ***S*** **. Enteritidis–Typhimurium–Infantis vaccine against challenge with homologous serovars.** NV: non-vaccinated chickens, V: vaccinated chickens, SE, STM and SI: challenge with *S*. Enteritidis, Typhimurium or Infantis, respectively. Left panel, log CFU/g of liver 4 dpi; right panel, log CFU/g of liver 14 dpi. * significantly different from the appropriate non-vaccinated control group by t test at *P* < 0.05.

When the chickens were challenged with wild type *Salmonella* Enteritidis, Typhimurium and Infantis, all chickens were positive in the cecum and since we experienced overgrowth of nalidixic acid microbiota, exact quantification was not possible. However, numerically lower *Salmonella* counts were always observed in the liver of vaccinated chickens, both 4 and 14 dpi. Due to having only six chickens per group, individual variation among chickens and the low virulence of the wild type *S*. Infantis, the protective effect was statistically significant only after challenge with the most virulent *S*. Enteritidis (Figure 1).

### Protection against heterologous challenge

When 12 chickens were examined for the presence of the vaccine strains on day 21, i.e. just before challenge, all 12 chickens were positive for *Salmonella* in the cecum and 7 out of 12 chickens were positive also in the liver. Out of 88 randomly picked colonies, 1 belonged to serovar Enteritidis, 50 to Typhimurium and 37 to serovar Infantis.

**Figure 2:**
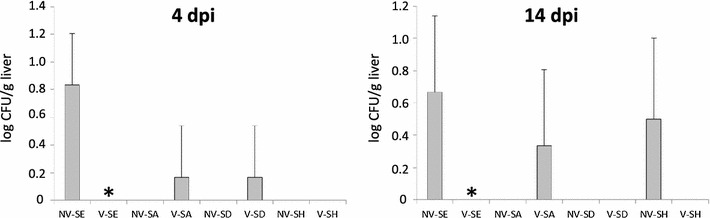
**Protective effect of** ***S*** **. Enteritidis–Typhimurium–Infantis vaccine against challenge with heterologous serovars.** NV: non-vaccinated chickens, V: vaccinated chickens, SE, SA, SD and SH: challenge with *S*. Enteritidis, Agona, Dublin and Hadar, respectively. Left panel, log CFU/g of liver 4 dpi; right panel, log CFU/g of liver 14 dpi. * significantly different from the appropriate non-vaccinated control group by t test at *P* < 0.05.

When the vaccinated chickens were challenged with wild type *S*. Agona, *S*. Dublin, *S*. Hadar and *S*. Enteritidis as a control, the vaccination did not affect *S*. Enteritidis and *S*. Agona presence in the cecum. However, vaccination decreased *S*. Dublin cecum colonization and nearly completely protected chickens against *S*. Hadar infection (Table 2). The higher number of *S*. Dublin positive chickens in the group of vaccinated birds than in the non-vaccinated chickens at 4 dpi likely represented only random variation among low level colonized birds (Table 2). Liver colonization by *S*. Agona, *S*. Dublin and *S*. Hadar was quite low even in the non-vaccinated chickens and no significant differences between the vaccinated and non-vaccinated chickens were recorded (Figure 2). As in the previous experiment (Figure 1), the vaccination protected chickens against liver colonization following *S*. Enteritidis challenge (Figure 2).

**Table 2:** **Number of** ***Salmonella*** **positive chickens in the ceca of vaccinated and non-vaccinated chickens following** ***S*** **. Agona,** ***S*** **. Dublin,** ***S*** **. Hadar and** ***S*** **. Enteritidis challenge**

	Non-Vacc.	Vaccinated	Non-Vacc.	Vaccinated
Challenge	4 dpi		14 dpi	
Enteritidis	6/6^a^	6/6	6/6	5/6
Agona	6/6	6/6	6/6	6/6
Dublin	3/6	5/6	6/6	2/6
Hadar	4/6	1/6	6/6	0/6^#^

^a^Number of *Salmonella* positive chickens/number of tested chickens.

^#^Significantly different from the appropriate non-vaccinated control by χ^2^ test with *P* < 0.05.

### Long term shedding of vaccine strains

**Figure 3:**
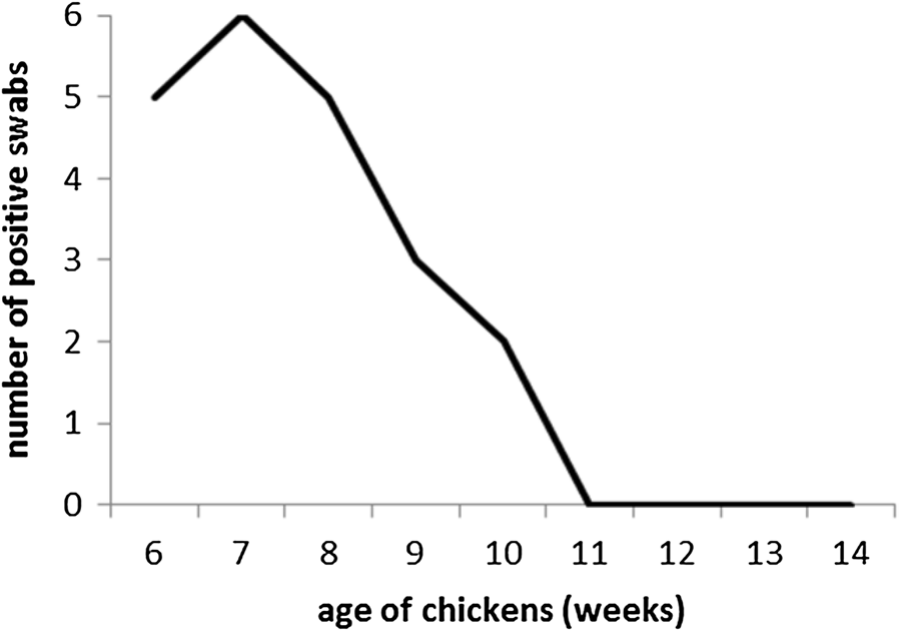
**Shedding of the vaccine strains by chickens as determined by cloacal swabbing.** The chickens were vaccinated on day 1, revaccinated on day 21 of life and *Salmonella* presence in cloacal swabs was detected until four consecutive negative tests were recorded.

Since we recorded that the vaccine strains were present in the vaccinated chickens even on day 21 of life, in the next experiment we determined fecal shedding of the vaccination strains. Six chickens were vaccinated on day 1 of life and revaccinated on day 21. Five or six out of six chickens were positive for *Salmonella* in cloacal swabs on weeks 6, 7 and 8 of life. A decrease in positivity was detected between weeks 9 and 11, and from week 11, all the chickens were *Salmonella* negative (Figure 3). Three individual isolates from three different chickens picked up on week 9 were confirmed by PCR to contain the SPI1 deletion, i.e. to be one of three possible serovars present in the vaccine. Serological testing showed that all these isolates belonged to serovar Infantis.

### Vaccination schemes and long term protection

In the last experiment we tested the long term protectiveness of the vaccine in three different vaccination regimes. Unlike previous experiments, the chickens were challenged with *S*. Enteritidis only, but both orally and intravenously, and inflammation in the cecum was determined to better characterize the immune response. The characterization of cecal inflammation also partially substituted for missing quantitative data on *Salmonella* in the cecum.

Although quantitative determination of *S*. Enteritidis in the cecum was difficult due to an overgrowth of nalidixic acid resistant microbiota, all the chickens, irrespective of age or mode of challenge, were positive for *Salmonella* in the cecum 4 dpi.

**Figure 4:**
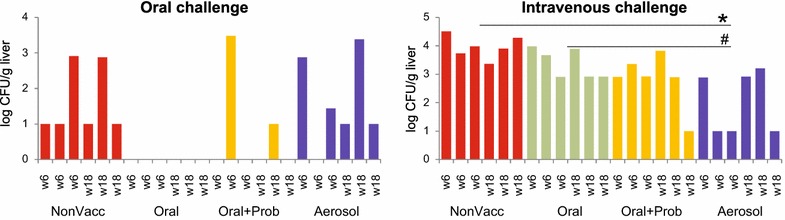
***S*** **. Enteritidis counts in individual chickens after oral or intravenous challenge.** Left panel, *S*. Enteritidis counts in the liver 4 days following oral challenge. “w” indicates age of individual chickens at the time of infection. Right panel, *S*. Enteritidis counts in the liver 4 days following intravenous challenge. * significantly different from non-vaccinated chickens, # significantly different from orally vaccinated chicken by ANOVA at *P* < 0.05.

Quantitative detection of *S*. Enteritidis in the liver showed that there were no differences between the response of 6 and 18-week chickens and we therefore combined data from these two age categories. Following the challenge, orally vaccinated and orally challenged chickens were free of *S.* Enteritidis in the liver (Figure 4). Despite this, comparison with *S*. Enteritidis counts in the liver of non-vaccinated or aerosol vaccinated chickens did not meet statistical significance (non-vacc vs. oral, *P* = 0.072, aerosol vs. oral, *P* = 0.076). Vaccination together with administration of cecal microbiota numerically reduced the efficacy of the vaccine but this difference did not reach statistical significance.

When the chickens were challenged intravenously, the best protection was observed in the chickens which were vaccinated by aerosol (Figure 4). Aerosol vaccinated chickens were significantly better protected against intravenous challenge than non-vaccinated or orally vaccinated chickens. Chickens vaccinated orally with cecal microbiota exhibited intermediate protection against intravenous challenge and the comparison of this group of chicken with non-vaccinated control approached statistical significance (non-vacc vs. oral + probio, *P* = 0.095).

**Figure 5:**
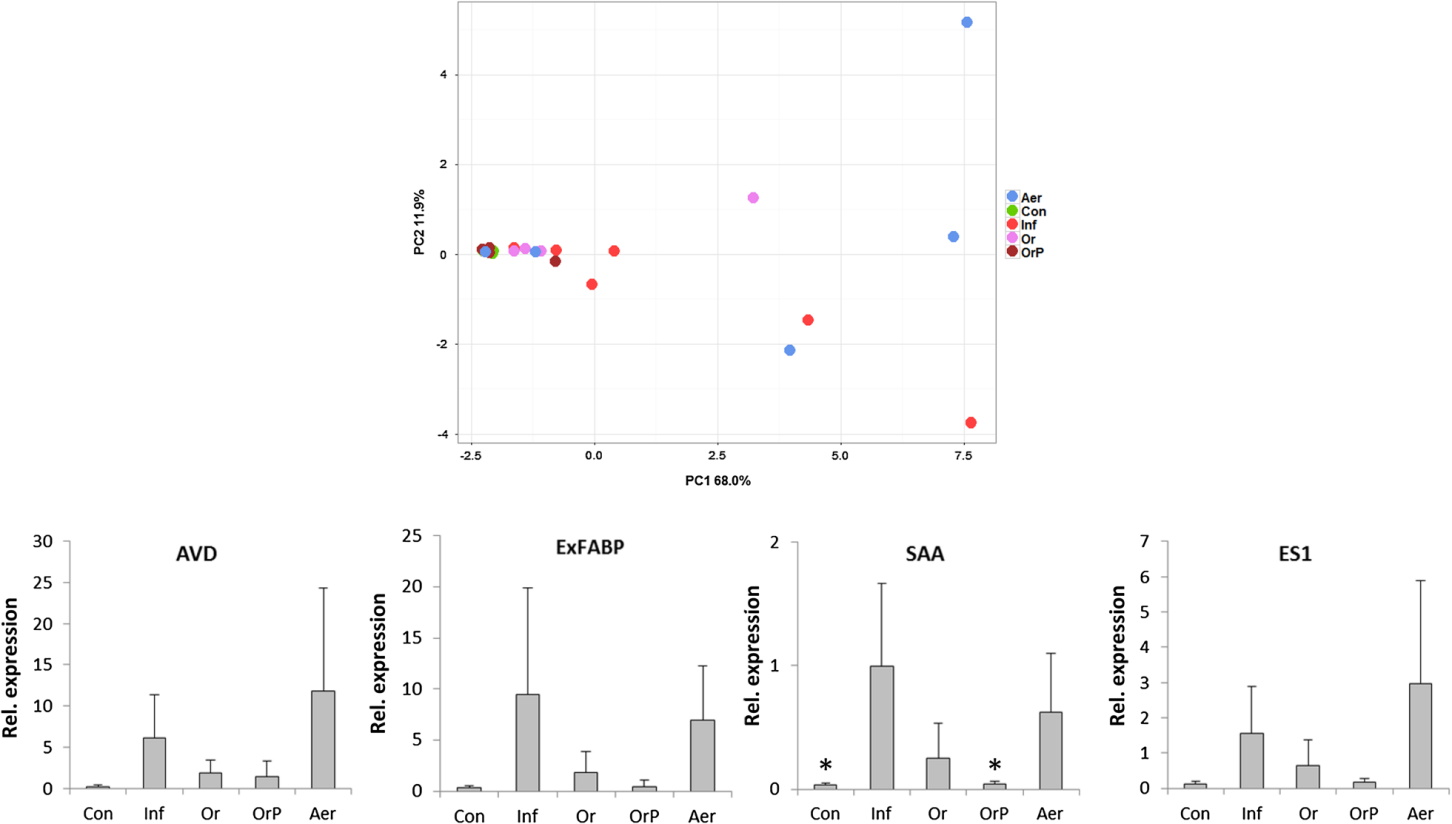
**Gene expression in the chicken in cecal tissue following vaccination and oral challenge.** Upper panel, Principal Component Analysis (PCA) of individual chickens using “integrated” expression of 16 genes used for the characterization of chicken response to *S*. Enteritidis challenge. The chickens vaccinated by aerosol responded to the infection similarly as the non-vaccinated chickens indicating the lowest protection. On the other hand, orally vaccinated chickens clustered with the non-infected controls showing the highest protective effect of oral vaccination. Con: control, non-vaccinated and non-infected chickens, Inf: non-vaccinated but infected chickens, Or: orally vaccinated and infected chickens, OrP: orally vaccinated together with administration of cecal microbiota (probiotics) and infected chickens, Aer: aerosol vaccinated and infected chickens. Lower row of four panels, average expression of four genes with the highest expression in the chicken cecum following oral infection with *S*. Enteritidis. AVD: avidin, ExFABP: extracellular fatty acid binding protein, SAA: serum amyloid A, ES1: ES1 protein homolog, mitochondrial-like. For the expression of all individual genes, see Additional file 1. * significantly different from non-vaccinated chickens but infected chickens by ANOVA at *P* < 0.05.

Finally we determined the inflammatory response in the cecum of orally challenged chickens and in the liver of intravenously challenged chickens. The inflammatory response was determined by the expression of 16 genes in the cecal tissue and 13 genes in the liver selected according to our previous reports [28, 29]. Expression of these genes was used only as a marker of inflammatory response expecting an increased response of naive animals to challenge with the wild type *S*. Enteritidis and lower or no response of vaccinated and challenged, or control chickens, respectively. We therefore did not consider biological function of these genes in this study. An increased inflammatory response in the cecum following oral challenge, similar to that in the non-vaccinated chickens but challenged chickens was observed in the chickens vaccinated via aerosol indicating quite low protection (Figure 5). On the other hand, effective protection characterized by a low inflammatory response, similar to that observed in the non-vaccinated and non-infected chickens, was observed in orally vaccinated groups of chickens. Due to the repeatedly lower inflammatory response to the challenge in the cecum of chickens administered the vaccine together with cecal microbiota in comparison with the chickens vaccinated without cecal microbiota, we concluded that such chickens were slightly better protected than the chickens vaccinated with the vaccine only (Figure 5).

**Figure 6:**
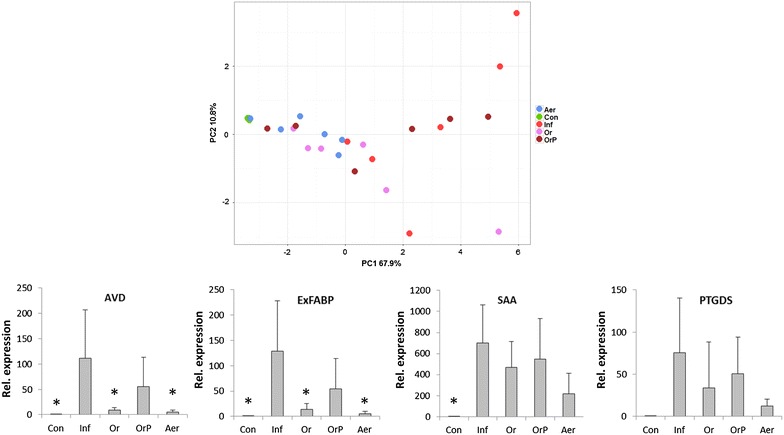
**Gene expression in the chicken liver following vaccination and intravenous challenge.** Upper panel, Principal Component Analysis (PCA) of individual chickens using “integrated” expression of 13 genes used for the characterization of chicken response to *S*. Enteritidis challenge. The chickens vaccinated with the vaccine mixed with probiotics responded to the infection similarly as the non-vaccinated chickens indicating their lowest level of protection. On the other hand, aerosol vaccinated chickens clustered the closest to the non-infected controls indicating the highest level of protection against intravenous challenge. Con: control, non-vaccinated and non-infected chickens, Inf: non-vaccinated but infected chickens, Or: orally vaccinated and infected chickens, OrP: orally vaccinated together with administration of cecal microbiota (probiotics) and infected chickens, Aer: aerosol vaccinated and infected chickens. Lower row of four panels, average expression of four genes with the highest expression in the chicken cecum following oral infection with *S*. Enteritidis. AVD: avidin, ExFABP: extracellular fatty acid binding protein, SAA: serum amyloid A, PTGDS: Prostaglandin D2 synthase. For the expression of all individual genes, see Additional file 1. * significantly different from non-vaccinated chickens but infected chickens by ANOVA at *P* < 0.05.

Gene expression in the liver after intravenous infection supported the results from bacteriology shown in Figure 4. A high inflammatory response to the challenge was observed in the non-vaccinated chickens (Figure 6). On the other hand, the most efficient protection characterized by a low inflammatory response characteristic of resistant chickens was observed in aerosol-vaccinated chickens. Unlike oral challenge, intravenous challenge resulted in repeatedly slightly higher inflammatory response in the liver of chickens given the vaccine together with cecal microbiota in comparison with the chickens vaccinated orally without cecal microbiota. This indicated that the cecal microbiota provided together with vaccine negatively affected development of systemic immunity (Figure 6).

## Discussion

In this study we compared the vaccine potential of *S*. Enteritidis, *S*. Typhimurium and *S*. Infantis SPI1, *lon* and flagella mutants in homologous and heterologous challenge models in chickens. In addition, we also compared aerosol and oral vaccine administration, and in the case of oral vaccination, we tested its administration together with cecal microbiota of adult hens.

Vaccine strains persisted in the chickens until approx. week 10 of life, the time at which also wild type *Salmonella* is eliminated [8]. This was rather unexpected since the non-invasive *hilA* mutant of *S*. Enteritidis, functionally similar to the SPI1 mutation used in the attenuated strains in this study, was reported to be shed for 4 weeks only [30]. This was probably caused by the presence of the *S*. Infantis strain in the vaccine, which persisted in the inoculated chickens the longest. However, when we used the parental *S*. Infantis strain for challenge, this strain was the least virulent, which was consistent with an earlier report on the virulence of *S*. *enterica* for chickens where *S.* Enteritidis was the most virulent, followed by *S.* Typhimurium, and lastly *S.* Infantis [31]. Despite the lower recovery of the *S*. Enteritidis vaccine strain, the vaccination protected against challenge with all serovars present in the vaccine. The vaccine exhibited a protective effect also towards *S*. Hadar and partial protection against *S*. Dublin. *S*. Enteritidis, *S*. Typhimurium and *S*. Infantis vaccine did not protect chickens against *S*. Agona. This was a little bit surprising since we expected cross-protection between *S*. Agona and *S*. Dublin due to the presence of the common O-antigen in *S*. Agona and *S*. Typhimurium, and in *S*. Dublin and *S*. Enteritidis. Our results therefore showed that vaccination with *S*. Enteritidis, *S*. Typhimurium and *S*. Infantis vaccine exhibited a certain degree of cross-protection against strains belonging to heterologous serovars but such protection was dependent not only on serovar classification but also on the overall genetic composition of each individual isolate. However, we have to remind that the differences following the challenge with heterologous serovars were quite low, both due to the increasing resistance of chickens to *Salmonella* infection with increasing age [32] and reduced virulence of non-*S*. Enteritidis serovars for chickens [31] and additional experiments will have to be performed to finally confirm or dispute these observations.

Aerosol vaccination is an efficient alternative to oral vaccination [10–12]. Another alternative increasing the vaccine’s potential is the administration of a live *Salmonella* vaccine together with probiotics or competitive exclusion products [16]. Both *Salmonella* counts and the inflammatory response confirmed that aerosol vaccination induced a greater systemic immune response. However, unlike in previous studies [10–12], the aerosol vaccination was less protective in the cecum than the oral vaccination. It is likely that multiple sites including the digestive tract, respiratory tract or conjunctiva are stimulated during aerosol vaccination which results in a systemic immune response. On the other hand, oral administration of the vaccine resulted mainly in a localized stimulation of the intestinal tract. These results were confirmed both by *Salmonella* counting and by determination of inflammatory response. Based on our previous reports we assumed that lower response after challenge with wild type *Salmonella* corresponded with higher protection [7, 27–29]. The response of individual chickens was similar for different gene categories like chemokine and cytokines (CSF3, AH221, SCYA4, IL-22, IL8, INFγ), acute phase proteins (ExFABP, MRP126, SAA, TRAP6) or proteins and enzymes with effector functions (IL4I1, iNOS, LYG2, IgG, PTGDS). Since the acute phase proteins are usually expressed at higher levels than cytokines and therefore provide more reliable results following real time PCR, we highlighted them in Figures 5 and 6.

Co-administration of cecal microbiota from adult healthy hens together with the vaccine did not improve vaccine efficacy in terms of reduced *Salmonella* counts in the liver, but slightly decreased inflammatory signaling in the cecum following oral challenge. Administration of microbiota and the vaccine resulted also in a lower systemic protection since higher *Salmonella* counts and higher inflammatory response were observed following intravenous challenge. The co-administration of the vaccine and microbiota might lead to decreased antigen recognition and development of a systemic and specific immune response. On the other hand, administration of healthy microbiota decreased inflammatory responsiveness after oral challenge though it will have to be tested whether a single microbiota administration on day 1 of life may affect inflammatory signaling at 6 or even 18 weeks of age. Despite this, co-administration of a live *Salmonella* vaccine together with undefined microbiota presents an interesting topic for future investigations, although we have to admit that the differences in the gene expression in the cecum were quite low, likely due to know increase in general resistance of chickens to colonization with *S*. Enteritidis or *S*. Typhimurium with increasing age [8, 32].

We have shown that a vaccine consisting of attenuated *S*. Enteritidis, *S*. Typhimurium and *S*. Infantis strains protects chickens against challenge with the wild type strains of the same serovars and may protect also against isolates belonging to other serovars such as Dublin or Hadar although protection against heterologous serovars may depend on the particular genetic composition of each field isolate. Aerosol vaccine administration is an interesting alternative to oral vaccination. However, care must be taken on the site of expected maximal immune protection. In addition, aerosol administration in particular may raise question on the vaccine safety to human personnel, an issue which we did not address in this study. Finally, an additional co-administration of microbiota from healthy adult hens together with the vaccine may (i) protect the chickens immediately after administration, (ii) induce a specific immune response following the vaccination, and (iii) even decrease the inflammatory responsiveness of adult hens. Administration of gut microbiota of appropriate composition may protect young chicken on its own, without induction of specific anti-*Salmonella* immune response, however, this may represent an issue in egg laying hens during egg laying period.

## Additional file

10.1186/s13567-016-0371-8 **Expression of all the genes tested in this study and included in the calculation of the PCA plots for individual chickens.** This file contains expressions of genes associated with chicken inflammatory response for each individual chicken. Expressions of slightly different subsets of genes were determined in the cecum or liver based on the maximal responsiveness of these genes in either of the tissues. Each line represents one chicken and expressions of genes normalized to average expression of GAPDH, TBP and UB.
